# Spatial transcriptomics identifies SPARC as a prognostic marker in interstitial lung diseases

**DOI:** 10.1002/path.6451

**Published:** 2025-07-28

**Authors:** Takayuki Niitsu, Toshiaki Kataoka, Kiyoharu Fukushima, Daisuke Motooka, Shigeyuki Shichino, Yayoi Natsume‐Kitatani, Hideya Kitamura, Takashi Niwa, Tomohisa Baba, Daisuke Okuzaki, Atsushi Kumanogoh, Shizuo Akira, Koji Okudela, Takashi Ogura

**Affiliations:** ^1^ Department of Respiratory Medicine and Clinical Immunology Osaka University Graduate School of Medicine Suita Japan; ^2^ Laboratory of Host Defense, World Premier Institute Immunology Frontier Research Center (WPI‐IFReC) Osaka University Osaka Japan; ^3^ Department of Pathology, Faculty of Medicine Saitama Medical University Saitama Japan; ^4^ Department of Infection Metagenomics, Genome Information Research Center, Research Institute for Microbial Diseases (RIMD) Osaka University Osaka Japan; ^5^ Division of Molecular Regulation of Inflammatory and Immune Diseases, Research Institute for Biomedical Sciences Tokyo University of Science Chiba Japan; ^6^ National Institutes of Biomedical Innovation Health and Nutrition Osaka Japan; ^7^ Institute of Advanced Medical Sciences Tokushima University Tokushima Japan; ^8^ Department of Respiratory Medicine Kanagawa Cardiovascular and Respiratory Center Yokohama Japan

**Keywords:** interstitial lung diseases (ILDs), spatial transcriptomics (ST), single‐cell RNA sequencing (scRNA‐seq), fibrosis, SPARC, unclassifiable ILDs (uILDs)

## Abstract

Interstitial lung diseases (ILDs) encompass a diverse group of pulmonary disorders, with progressive fibrosis leading to poor prognosis. Here we aimed to identify key molecules involved in progressive fibrosis across various ILDs, using spatial transcriptomics (ST). ST analysis (Visium) was performed on lung cryobiopsy specimens from five patients with various ILDs. Two cases, rich in young fibrotic lesions, as defined by fibroblastic foci and destructive alveolar organization, were selected for spatial high‐dimensional weighted gene coexpression network analysis (hdWGCNA) to identify key gene networks with biological significance in active fibrosis. We utilized public single‐cell RNA sequencing datasets of various ILDs, performed enrichment analysis and trajectory‐based differential expression analysis, and quantified cell–cell communication to evaluate the involvement of the spatially extracted module in fibrosis. Immunohistochemical staining of the extracted molecules was performed. Using hdWGCNA, we identified a distinct gene module (the SM2 module) enriched in young fibrotic lesions. The SM2 module was characterized by distinct features of fibroblast activation that were represented across various lesions. Key hub genes within this module, including *COL1A2*, *COL3A1*, *COL1A1*, and *SPARC*, formed a robust coexpression network. Immunohistochemical staining showed that SPARC, a component of the SM2 module, was highly expressed in young fibrotic lesions, but not in old scarring lesions, across various ILDs. To assess the prognostic significance of SPARC immunohistochemical expression, we extended our analysis to a cohort of 71 patients with unclassifiable ILDs (uILDs), a particularly heterogeneous subtype with unclear pathogenesis and limited treatment options. Higher SPARC levels in the upper, lower, or both lung lobes in uILD were significantly associated with poor overall survival. In summary, an integrated cross‐disease approach using ST revealed key gene expression patterns central to active fibrosis and successfully identified SPARC as a potentially beneficial prognostic marker. © 2025 The Author(s). *The Journal of Pathology* published by John Wiley & Sons Ltd on behalf of The Pathological Society of Great Britain and Ireland.

## Introduction

Interstitial lung diseases (ILDs) encompass over 200 distinct pulmonary disorders affecting the tissue and spaces around the alveoli [1]. Patients with ILDs showing progressive fibrosis have a poor prognosis [2]; however, the underlying mechanisms remain poorly understood. Consequently, no pharmacological treatments can fully reverse or eliminate the disease [3, 4]. Moreover, ILDs exhibit considerable heterogeneity, both among patients and across disease types [5], complicating the ability to predict progression to pulmonary fibrosis and the development of objective clinical strategies.

Recent advances in single‐cell RNA sequencing (scRNA‐seq) have revealed various factors involved in the pathogenesis of fibrosis [6]. Furthermore, the emergence of spatial transcriptomics (ST) now enables comprehensive characterization of the molecular landscape, while preserving the spatial context of tissue samples. This approach facilitates the identification of pathological cell populations within fibrotic niches [7].

However, a significant gap remains in our understanding of the shared and distinct molecular mechanisms driving fibrosis across heterogeneous ILD cohorts. To date, no studies have used ST to identify key molecules relevant to disease activity and prognosis across the different types of ILDs. Establishing prognostic universal biomarkers for ILDs could facilitate more timely and appropriate therapeutic interventions, potentially improving patient outcomes.

A recent hypothesis suggests that the fibrotic process, irrespective of its underlying etiology, shares common downstream pathways that lead to fibroblast activation and self‐perpetuating fibrosis [8].

Therefore, we aimed to explore common and essential molecules that promote the fibrotic process across disease types using multimodal ST analyses and to evaluate the clinical relevance of identified molecular markers, potentially establishing prognostic indicators for this challenging subgroup.

## Materials and methods

### Ethics approval and patient consent

This study was approved by the Ethics Committees of Yokohama City University (A180726009) and Kanagawa Prefectural Cardiovascular Respiratory Center Hospital (KCRC‐19‐0015). Informed consent was obtained from all patients for the use of resected materials for research purposes.

### Visium spatial transcriptomics: image acquisition and sequencing

Spatial transcriptomic profiling was performed using the Visium Spatial Gene Expression platform (10x Genomics, San Francisco, CA, USA) on formalin‐fixed paraffin‐embedded tissue sections of five lung cryobiopsy specimens from patients with various ILDs. The specimens retained histological and morphological integrity, with DV200 values (percentage of RNA fragments >200 nucleotides) greater than 50%. Libraries were sequenced on the DNBSEQ‐G400RS (MGI Tech, Shenzhen, Guangdong, PR China) in paired‐end mode (read 1:28 bp; read 2:50 bp), and sequence reads were processed using Space Ranger software (v. 1.3.0; 10x Genomics) [https://www.10xgenomics.com/support/jp/software/space-ranger/latest; last accessed 15 February 2022].

### Visium spatial transcriptomics: image analysis

Alignment of reads to the reference genome was performed using STAR (Spliced Transcripts Alignment to a Reference) [9], followed by filtering to remove duplicates and low‐quality reads. The filtered reads were then assigned to specific spatial barcodes based on their corresponding spot coordinates on the Visium slide. Gene expression levels were quantified by counting unique molecular identifiers associated with each gene within each spatially resolved tissue spot.

### Weighted gene coexpression network analysis in high‐dimensional transcriptomics data

Spatial transcriptomic data from the Visium platform were analyzed using a high‐dimensional extension of the standard WGCNA (hdWGCNA) approach to accommodate the increased complexity and volume of spatial transcriptomic data [10]. Genes expressed in at least 5% of the dataset were selected for analysis. Using the TestSoftPowers function in the R hdWGCNA package (https://smorabit.github.io/hdWGCNA/articles/ST_basics.html; last accessed 15 May 2025), a soft threshold of six was selected as optimal, yielding a network topology most consistent with biological relationships. Relationships between gene modules within the coexpression network were assessed by measuring gene expression similarity, calculating the topological overlap matrix, and performing hierarchical clustering analysis. The resulting hierarchical structure of the coexpression network was visualized using a dendrogram. Based on these results, dotplots were generated to represent the modules and module‐specific genes.

The hdWGCNA lacks a standardized approach for selection of young fibrotic lesions for comparison. Due to the limited resolution, avoiding the inclusion of pathologically dispersed cell populations is critical [11], as this risks introducing noise by incorporating gene profiles from unrelated cell populations. Therefore, we designated comparator spots without ILD‐related pathological changes as the ‘area without ILD‐related pathological changes’ for comparison.

Visium ST datasets from ILD samples were analyzed using the Seurat package in R (v. 4.4.2). Datasets were merged, normalized with SCTransform, and integrated using the Seurat pipeline and Harmony [12] (https://github.com/immunogenomics/harmony; last accessed 6 February 2025) for batch effect correction. PCA, UMAP, and spatial feature plots were used for clustering and visualization.

### Patients and lung tissues for histopathological examination

A total of 305 patients with ILDs who underwent surgical lung biopsy (SLB) at Kanagawa Cardiovascular and Respiratory Centre (Yokohama, Japan) between January 2008 and May 2019 were examined. We included 61 cases of idiopathic pulmonary fibrosis (IPF), 173 cases of non‐IPF, and 71 cases of unclassifiable ILD (uILD) (equivalent to unclassifiable idiopathic interstitial pneumonia), respectively. The patients did not receive any treatment for ILDs before undergoing a biopsy. The median follow‐up period was 1650 days (range, 6–4422 days).

### Disease subtyping

Final diagnoses were reached through a multidisciplinary discussion (MDD) based on clinical, radiological, and pathological findings. Here, three pathologists, two radiologists, and three respirologists participated and decided on disease types according to the official ATS/ERS/JRS/ALAT 2018 clinical practice guidelines [13]. In brief, the MDD process was structured as follows [14]:

Step 1: Deidentified clinical data were presented in a standardized format.
Step 2: High‐resolution CT (HRCT) findings, interpreted by the radiologist, were incorporated.
Step 3: Specific histopathological patterns, guided by consensus recommendations and derived from either transbronchial lung cryobiopsy or SLB, were introduced by the pathologists.

Following Step 2, MDD participants independently recorded their provisional diagnoses along with their diagnostic confidence. Subsequently, a moderated discussion was conducted to establish a consensus diagnosis, a set of potential differential diagnoses, and an associated level of diagnostic confidence. Confidence levels were categorized as ‘definite’ (90%–100%), ‘high’ (70%–89%), or ‘low’ (51%–69%), based on the ontology [15]. In cases deemed ‘unclassifiable,’ no confidence level was assigned.

After Step 3, participants again documented their diagnoses and confidence levels. This was followed by determining a final consensus diagnosis and its corresponding confidence level.

For uILD classification, when no diagnosis exceeded 50% confidence, the case was classified as ‘uILD’ [16]. The reasons for remaining unclassifiable included insufficient clinical, radiological, or pathological data, inadequate histopathological sampling, or atypical/overlapping features not meeting diagnostic criteria [17].

The diagnostic proportions at our institution (Kanagawa Cardiovascular and Respiratory Centre) were comparable to those in previously published studies [18, 19], suggesting a high degree of diagnostic accuracy.

### Conventional histopathological examination

Lung tissues were fixed in 10% buffered formaldehyde solution for ~24 h and embedded in paraffin wax. Tissue sections were stained using hematoxylin and eosin (HE): hematoxylin (Merck KGaA, Darmstadt, Germany) and eosin (HE) (Waldeck GmbH & Co. KG, Division Chroma, Munster, Germany); Elastica van Gieson (EVG): Resorcin‐fuchsin (Muto Pure Chemicals Co. Ltd., Tokyo, Japan), Sirius Red (Waldeck GmbH & Co. KG), picric acid (Fujifilm Wako Pure Chemical Corporation, Osaka, Japan); Alcian blue–Periodic Acid Schiff (AB‐PAS): Alcian blue (Fujifilm Wako Pure Chemical Corporation), Schiff (Muto Pure Chemicals Co. Ltd).

### Immunohistochemistry

Immunohistochemistry was performed using the Histostainer system (Nichirei Biosciences Inc., Tokyo, Japan). In brief, 5‐μm‐thick paraffin‐embedded tissue sections were incubated with hydrogen peroxide (Fujifilm Wako Pure Chemical Corporation) to inhibit endogenous peroxidase, following by blocking solution (Merck KGaA) to prevent nonspecific protein binding. The blocked tissue sections were then boiled in antigen retrieval buffer (Heat Processor Solution, pH 9.0, Nichirei Biosciences Inc.). Sections were incubated with the following primary antibodies: SPARC (secreted protein acidic rich in cysteine, AF941; R&D Systems, Minneapolis, MN, USA) at 1:250 dilution; COL1A1 (66948S; Cell Signaling Technology, Danvers, MA, USA) at 1:200 dilution; COL1A2 (ab270994; Abcam, Ltd, Cambridge, UK) at 1:2,000 dilution; COL3A1 (NB600‐594SS; Novus Biologicals, Inc., Centennial, CO, USA) at 1:200 dilution. This was followed by incubation with horseradish peroxidase‐labeled anti‐mouse immunoglobulin secondary antibody (Nichirei Biosciences Inc.). Immunoreactivity was visualized using diaminobenzidine (Agilent Technologies Japan, Ltd., Tokyo, Japan) as the substrate. Nuclei were lightly counterstained using hematoxylin.

### Semiquantitative analysis of SPARC immunohistochemical expression

The 71 patients diagnosed with uILD were investigated to elucidate the relationship between SPARC‐stained areas and prognosis. The median follow‐up period was 1,562 days (range, 6–5,189 days). During the follow‐up period, 12 patients died of ILD‐related respiratory failure. Patients with IPF (*n* = 18), non‐specific interstitial pneumonia (NSIP) (*n* = 8), fibrotic hypersensitivity pneumonitis (fHP) (*n* = 10), and systemic sclerosis (SSc)‐related ILD (*n* = 8), respectively, all diagnosed by multidisciplinary discussion at Kanagawa Prefectural Cardiovascular Respiratory Center Hospital between 2015 and 2019, were investigated to determine the relationship between ILD subtype and SPARC‐positive area. Upper‐ and lower‐lobe lung resection biopsy specimens from all patients were used.

The SPARC‐immunostained glass slides were scanned using a virtual NanoZoomer slide scanner (Hamamatsu Photonics, Hamamatsu, Japan). Positive areas (areas with signal over the threshold) were measured using the open‐source digital image analytical software Qu‐Path (https://qupath.github.io/) [last accessed 6 February 2025] [20]. The rate of SPARC‐positive area per section (SPARC rate) was calculated.

### Statistical analyses

We determined a cutoff value that maximizes the Youden index [21] using the receiver operating characteristic (ROC) curve between the SPARC‐positive rate and death events due to ILD exacerbation. Based on this cutoff value, patients were divided into high and low groups. Kaplan–Meier survival curves were generated for these groups, and differences between the curves were analyzed using the log‐rank test. Multivariate analyses were conducted using a generalized linear model to evaluate the association between SPARC‐positive rates and survival outcomes, adjusting for potential confounding factors, such as age, sex, and smoking history. Differences were considered statistically significant at *p* < 0.05. All statistical analyses were performed using JMP software (v. 16; SAS Institute Inc., Cary, NC, USA).

## Results

### Spatial coexpression network analysis uncovered the molecular landscape within the fibrotic lesion associated with poor prognosis

**Figure 1:**
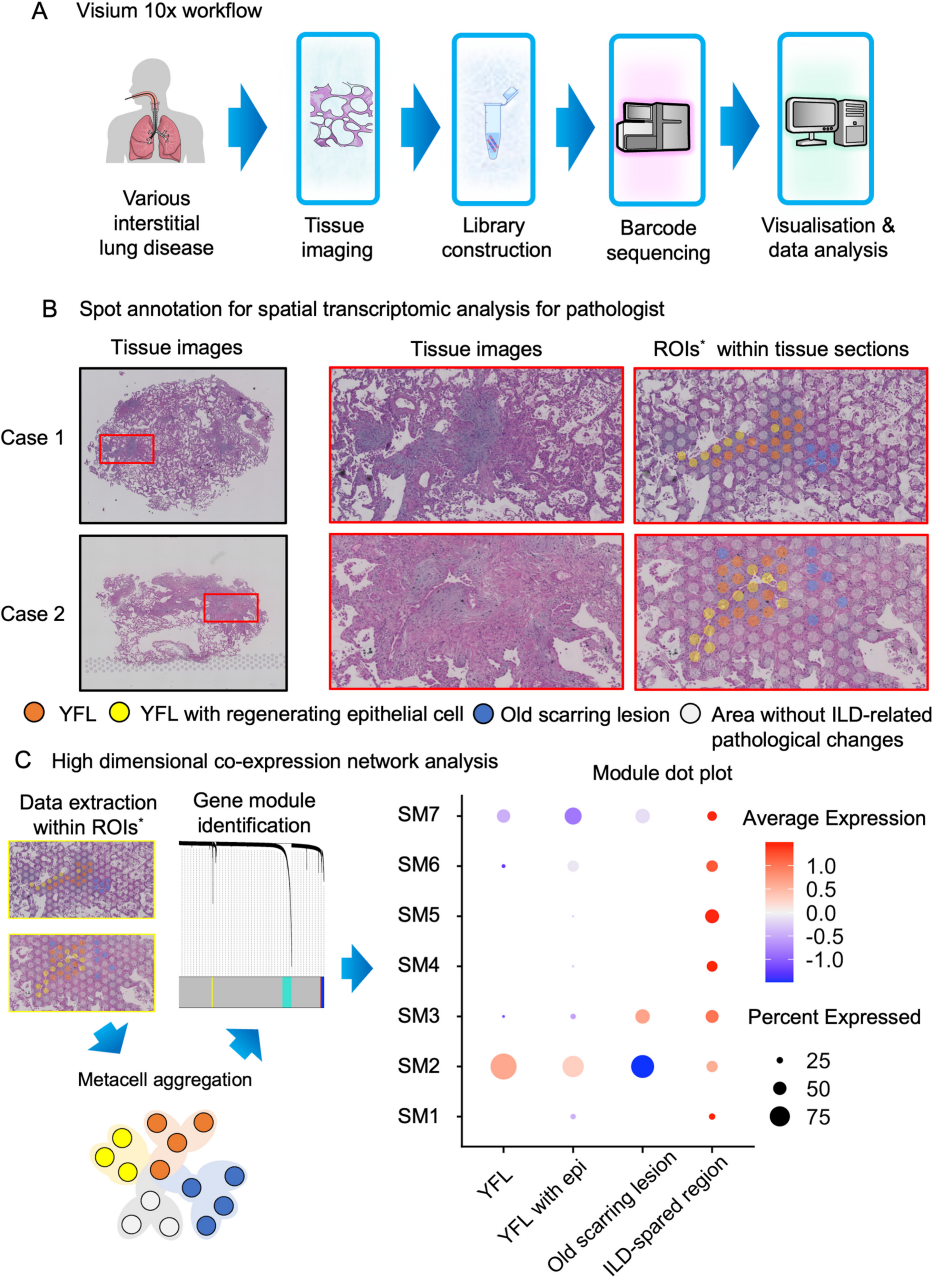
Spatial transcriptomics (ST) workflow and spatial coexpression analysis. (A) Summary of the ST workflow. (B) Spot annotation for ST in two cases rich in young fibrotic lesions (YFLs). Squares in the left panels (Cases 1 and 2) are magnified in the center and right panels. Fibroblastic aggregation involving a few alveoli (destructive alveolar organization) is observed in case 1, while fibroblastic aggregation at the lobular periphery (fibroblastic focus) is observed in case 2 (center panels). Region of interests (ROIs) were categorized based on fibrotic activity and cellular components into: YFLs; YFLs with regenerating epithelial cells; old scarring lesions; and areas without interstitial lung disease (ILD)‐related pathological changes. All panels were stained with hematoxylin and eosin (HE). (C) Integrative data synthesis from ROIs using high‐dimensional weighted gene coexpression network analysis (hdWGCNA). hdWGCNA was applied to spatial transcriptomic data to identify spatial modules (SMs). Gene expression data were extracted from ROIs and aggregated into metacells to reduce dimensionality. Coexpression modules (SM1–SM7) were defined based on gene–gene correlation structures across spatial locations. A module dotplot shows the average expression (color scale) and percentage of metacells expressing each module (dot size) across the four ROI categories: YFLs; YFLs with regenerating epithelial cells; old scarring lesions; and ILD‐spared regions (areas without ILD‐related pathological changes). Distinct modules exhibited YFL‐specific expression patterns.

We performed ST analysis (Visium, 10x Genomics) on five lung cryobiopsy specimens from patients with various ILDs, ensuring preserved histology and morphology with DV200 values >50% (Figure 1A). We focused on young fibrotic lesions, including fibroblastic foci, defined as fibroblastic aggregation covering alveolar collapse at lobular peripheries [22], and destructive alveolar organization, characterized by fibroblastic aggregation involving a few adjacent alveoli, resulting in their collapse [23], as these lesion types have been reported to correlate with disease progression and poor prognosis [24, 25]. Fibroblastic lesions were detected on HE‐stained histological sections with supplementary use of AB‐PAS and EVG stain (see supplementary material, Figure S1). For detailed analyses, we selected two cases that were particularly rich in young fibrotic lesions.

First, we histopathologically categorized regions of interest (ROIs) into four types (young fibrotic lesions; young fibrotic lesions with regenerating epithelial cells; old scarring lesions; and areas without ILD‐related pathological changes) (Figure 1B). We annotated the four types on the ST‐analyzed tissue sections and extracted gene expression profiles from the corresponding ROIs.

Next, we performed multimodal coexpression network analysis, hdWGCNA (Figure 1C), to identify gene modules related to young fibrotic lesions. This method facilitated the identification of robust gene–gene correlations with reduced noise by grouping multiple spots with similar gene expression profiles using a bootstrap aggregation approach. Rather than analyzing gene–gene correlations at the single‐cell level, this technique clusters similar expression patterns across spatially defined areas, allowing for a more stable and biologically relevant identification of key gene networks [10].

Finally, we identified distinct marker genes (SM2 module) that were enriched in young fibrotic lesions (Figure 1C). Furthermore, gene ontology (GO) pathway enrichment analysis indicated that the SM2 module encompasses biological processes essential for fibrosis, such as extracellular matrix (ECM) organization (GO:0030918) and platelet‐delivered growth factor binding (GO:0048407) (supplementary material, Figure S2).

In summary, hdWGCNA spatially identified gene modules related to young fibrotic lesions while preserving the essential fibrotic process within ROIs.

### Identification of key hub genes in young fibrotic lesions

**Figure 2:**
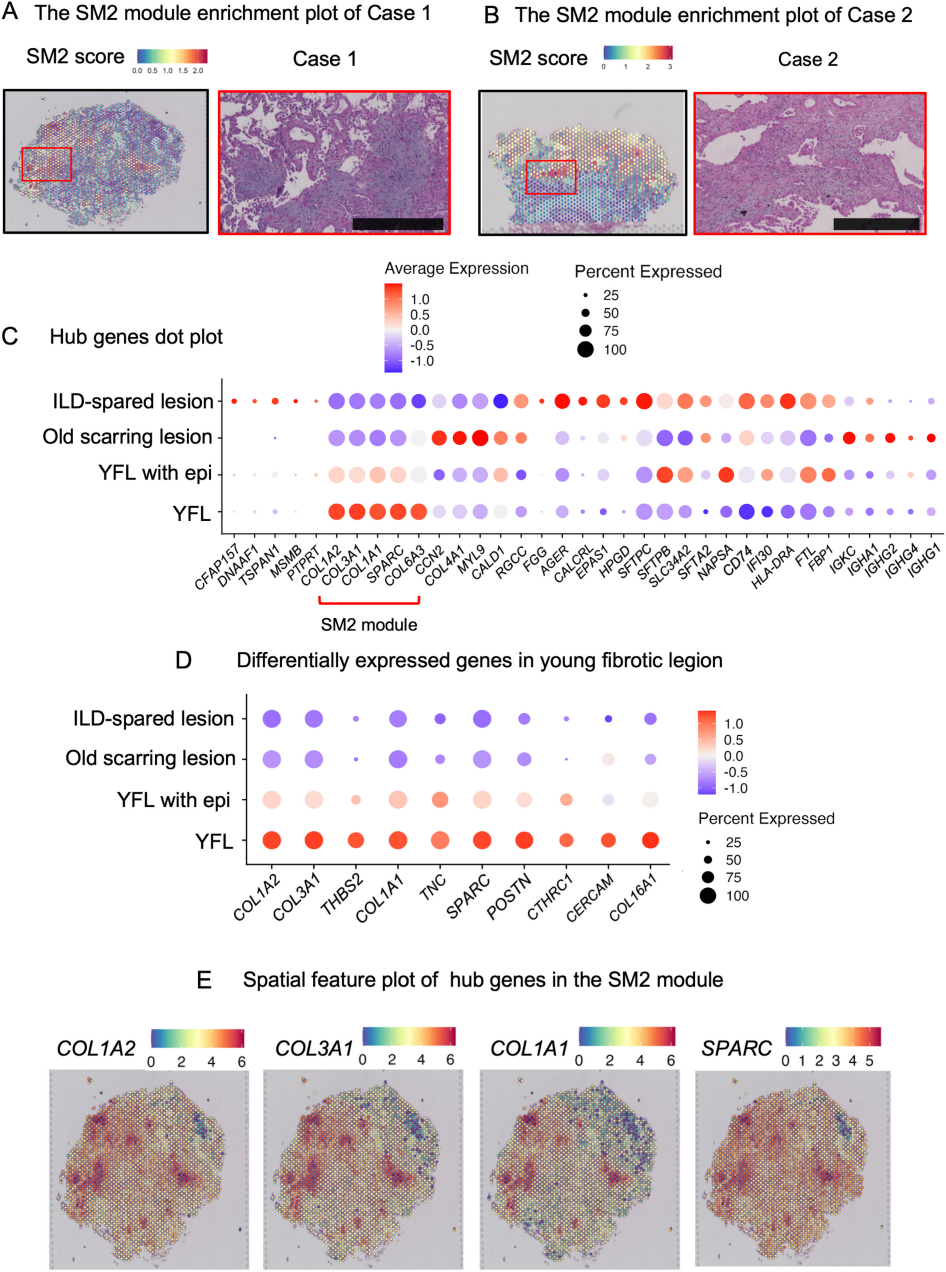
Spatial and network mapping of SM2 module and hub genes in ILD tissues. (A) Spatial feature plot mapping of the SM2 module in Case 1. The square in the left panel, where a high SM2 score is observed, is magnified in the right panel. Fibroblastic aggregation involving a few alveoli (destructive alveolar organization) is evident. Scale bars, 500 μm. (B) Spatial feature plot mapping of the SM2 module in Case 2. The square in the left panel, where a high SM2 score is observed, is magnified in the right panel. Fibroblastic aggregation at the lobular periphery (fibroblastic focus) is seen. All panels are stained with HE. (C) Dotplot of representative hub genes in the SM2 module across four pathological regions: YFLs, YFLs with regenerating epithelial cells, old scarring lesions, and ILD‐spared regions (areas without ILD‐related pathological changes). (D) Dotplot showing differentially expressed genes enriched in YFL compared to other regions. Several SM2 module hub genes are preferentially expressed in YFLs. (E) Spatial feature plots of representative SM2 hub genes (*COL1A2, COL3A1, COL1A1*, and *SPARC*) in Case 1. These genes show localized enrichment patterns corresponding to YFLs.

We performed enrichment analysis in two selected cases. We found that the SM2 module was specifically enriched in young fibrotic lesions (Figure 2A,B). To identify key genes playing a central role in the module, we calculated the module eigengene‐based connectivity (kME), which represents the correlation between each gene and the overall expression pattern of the module. Based on the kME values, we extracted the top hub genes with the highest connectivity [10].

The results showed that *COL1A2, COL3A1, COL1A1*, and *SPARC* were the top hub genes (Figure 2C). These genes were also identified as differentially expressed genes specific to young fibrotic lesions (Figure 2D). Spatial plots of the four genes showed consistent results (Figure 2E). These insights suggest that within the SM2 module, key genes such as *COL1A2, COL3A1, COL1A1*, and *SPARC*, form a robust network and are coexpressed in young fibrotic lesions.

### The SM2 module identifies young fibrotic lesions across ILD diseases

**Figure 3:**
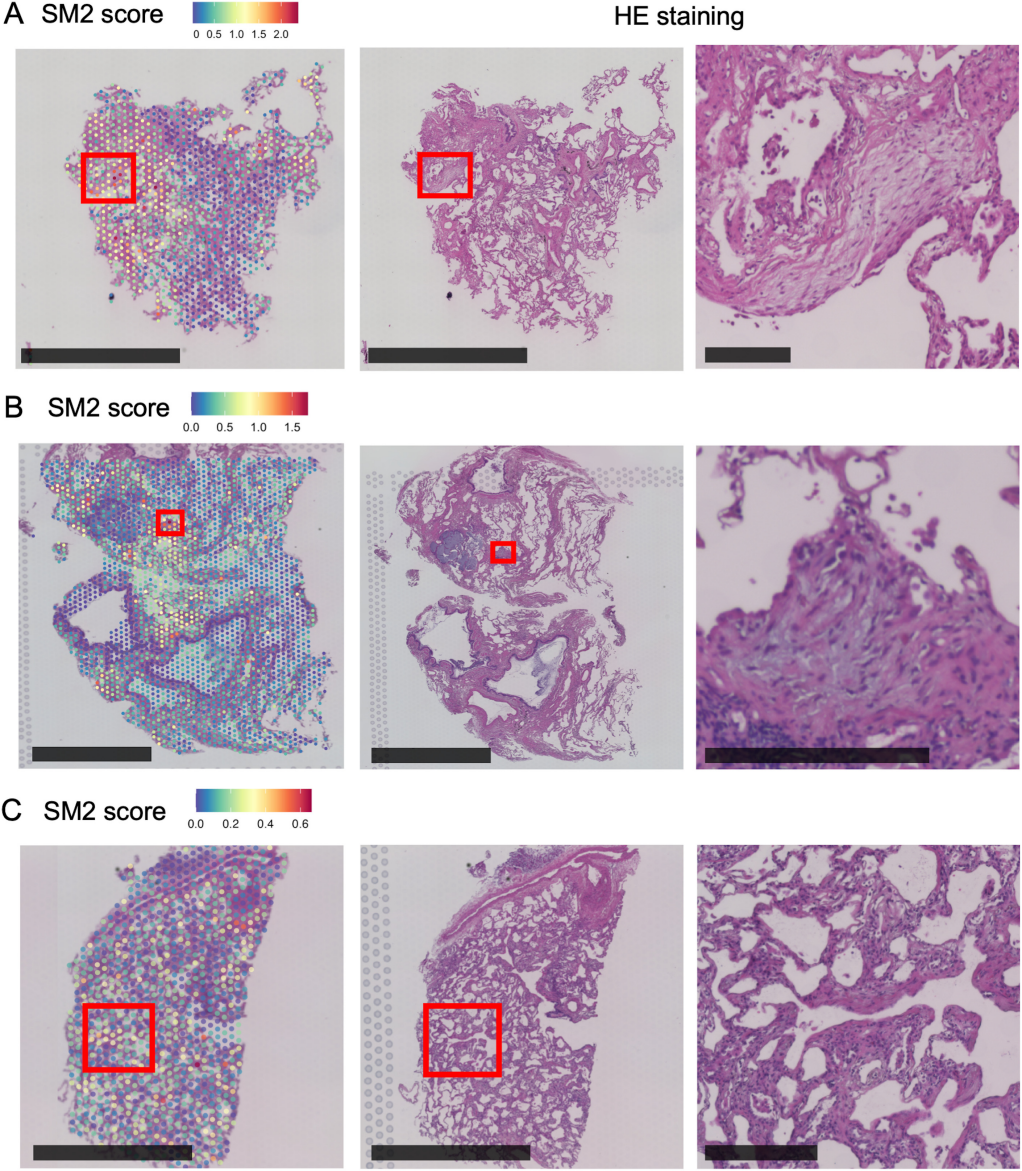
Example of SM2 module enrichment plot validation. Left panels show whole images of SM2 module enrichment plots; center panels show corresponding histological photographs (HE); and squares in the center panels are magnified in the right panels. The spots in squares (A) and (B) show high SM2 scores. The closer view in panel (A) shows a destructive alveolar organization in which fibroblastic cells aggregate involving a few alveoli, and that in panel (B) shows a fibroblastic focus (FF) in which fibroblastic cells aggregate sharing a lobular periphery. However, panel (C) shows a lower SM2 score with no young fibrotic lesion observed. Scale bars, 2 mm (left), 2 mm (center), 200 μm (right).

To examine whether common molecular mechanisms are shared across heterogeneous lesions, we investigated whether the SM2 module specifically highlights young fibrotic lesions within their histopathological context by analyzing its enrichment. The SM2 module showed prominent enrichment in specific regions corresponding to young fibrotic lesions (Figure 3A,B), while its expression was low in other regions (Figure 3C). These findings suggest that the SM2 module selectively identifies young fibrotic lesions, suggesting its potential utility in their detection.

### SM2 modules implicate fibroblast activation in ST

To explore which lung cell types were enriched in the SM2 module, we further assessed their potential role in the fibrotic process at the single‐cell level. We integrated the SM2 module into publicly available scRNA‐seq data from patients with different forms of pulmonary fibrosis (PF), including IPF (*n* = 12), cHP (*n* = 3), nonspecific interstitial pneumonia (NSIP) (*n* = 2), uILD (*n* = 1), and nonfibrotic controls (*n* = 10) (GSE135893; https://www.ncbi.nlm.nih.gov/geo/query/acc.cgi?acc=GSE135893) [26]. The SM2 module was enriched in the fibroblastic cell lineage, including Fibroblasts, HAS1 High Fibroblasts, Myofibroblasts, and PLIN2+ Fibroblasts (supplementary material, Figures S3A–C), with Myofibroblasts and HAS1 High Fibroblasts being the specific cell types most prominently enriched (supplementary material, Figure S3D,E). These cell populations were specific to patients with PF in this dataset, and HAS1 High Fibroblasts have previously been proposed to represent an intermediate differentiation state between quiescent fibroblasts and myofibroblasts [27].

Next, to determine whether the SM2 module was merely enriched in the activated fibroblasts or also dynamically along their differentiation trajectory, we employed tradeSeq to analyze gene expression changes across pseudotime trajectories [28]. The results demonstrated distinct lineages with varying gene expression patterns. Lineage 1 progressed toward Myofibroblasts (*CTHRC1* and SM2‐enriched cell type), Lineage 2 toward HAS1 High Fibroblasts (SM2‐enriched), and Lineage 3 toward Myofibroblasts (quiescent marker‐enriched (*INMT, NPNT*)) (supplementary material, Figure S4A,B). Furthermore, the top hub genes of the SM2 module—*COL1A1, COL1A2, COL3A1*, and *SPARC—*showed upregulation along the trajectories leading toward Lineage 2 and Lineage 3 (supplementary material, Figures S4D,E and S3C–E).

These findings are consistent with the earlier spatial transcriptomic results described in the SM2 module identification section (Figure 2C,D), which demonstrated that *COL1A1, COL1A2, COL3A1*, and *SPARC* are specifically associated with young fibrotic lesions.

### Fibroblasts highly expressing top hub genes potentially interact with aberrant basaloid cells

To investigate which cell type interacts with fibroblast populations that highly express the top hub genes of the SM2 module, we performed CellChat analysis [29]. The analysis revealed that HAS1 High Fibroblasts and Myofibroblasts exhibited a central role in intercellular communication with KRT5−/KRT17+ aberrant basaloids, which express fibrosis‐ and senescence‐associated genes [6] (supplementary material, Figure S5A,B). Key signaling pathways targeting KRT5−/KRT17+ aberrant basaloids indicated significant enrichment of the collagen signaling pathway, predominantly driven by HAS1 High Fibroblasts and Myofibroblasts (supplementary material, Figure S5C). In the collagen signaling pathway, strong interactions were observed for key genes, including *COL1A1* and *COL1A2*, which were predominantly linked to Myofibroblasts and HAS1 High Fibroblasts. Integrins, along with *SDC1* and *CD44*, were identified as major receptors (supplementary material, Figure S5D). These findings suggest that fibroblasts expressing the top hub genes play a pivotal role in intercellular communication and in regulating fibrosis through collagen‐associated signaling pathways.

### SPARC is specifically expressed in young fibrotic lesions

**Figure 4:**
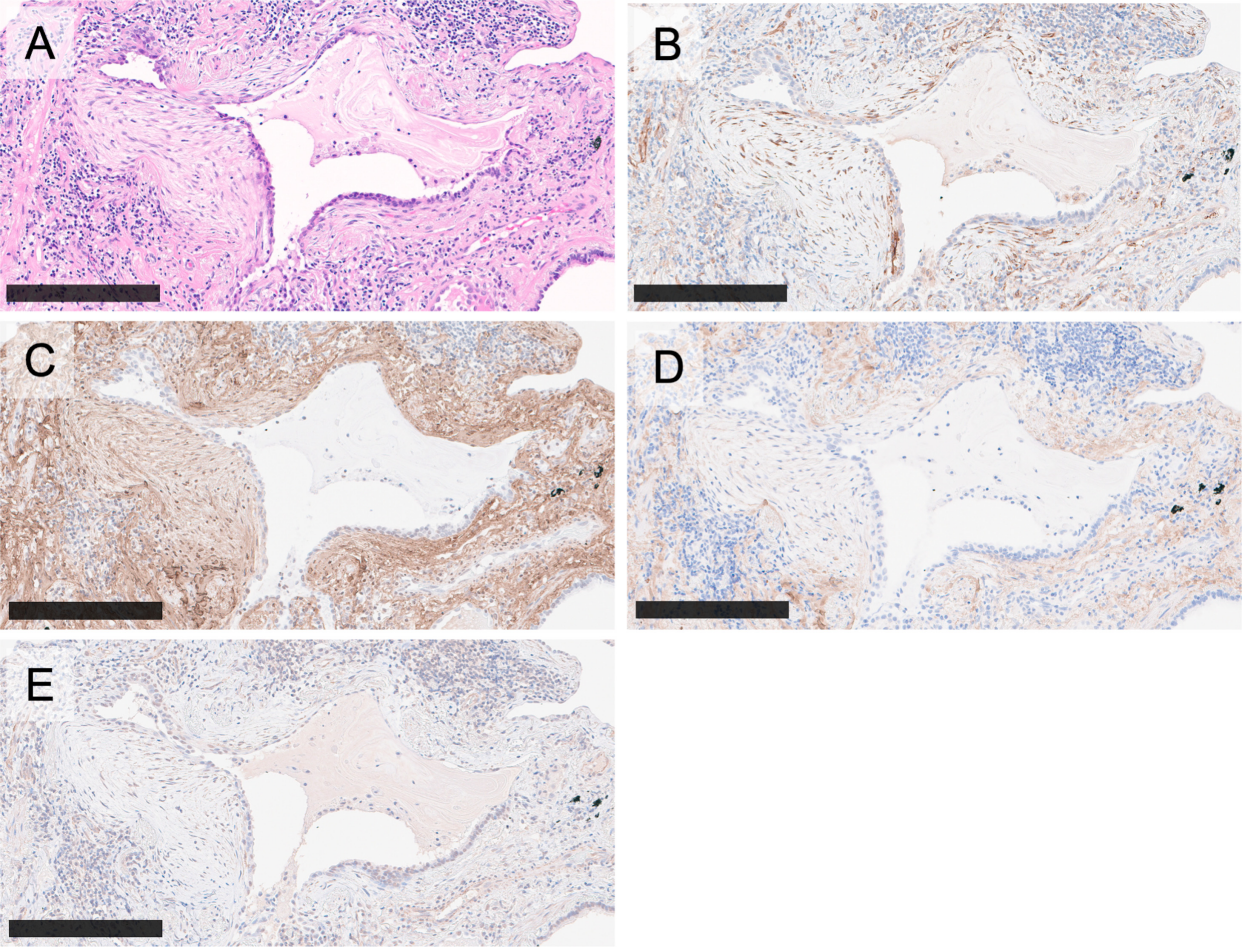
Immunohistochemical expression of secreted protein acidic and rich in cysteine (SPARC) and collagen genes (COL1A1, COL1A2, and COL3A1) in young fibrotic lesions. Histological sections of (A) HE staining of a young fibrotic lesion covering old scarring (fibrotic focus [FF]). (B) Strong SPARC expression in the cytoplasm of fibroblastic cells within the FF. (C) diffuse COL1A1 expression in both the FF and surrounding old scarring. (D) Stronger COL1A2 expression in the old scarring compared to the FF. (E) Diffuse and faint COL3A1 expression. Scale bars, 250 μm.

Immunohistochemistry for the three SM2 hub genes/proteins was performed to further confirm the results from the ST analyses and identify the best biological marker to detect young fibrotic lesions in pathological examinations. SPARC signals were strongly and evidently seen in young fibrotic lesions (Figure 4A,B). In contrast, COL1A1 and COL1A2 signals were weak and diffuse throughout the lung interstitial tissue (Figure 4C,D). COL3A1 signals were prominently observed in fibroblastic foci, but they were not entirely specific, as signals were also detected in the alveolar epithelium, vascular endothelium, smooth muscle, and certain lymphocytes (Figure 4E). These findings suggest that SPARC may serve as the most reliable biological marker.

Based on the results of the scRNA‐seq analysis with various ILDs, SPARC was suggested to be highly expressed not only in Myofibroblasts but also in intermediate fibroblast cell types, HAS 1 High fibroblasts, during the process of fibroblast activation across diseases.

**Figure 5:**
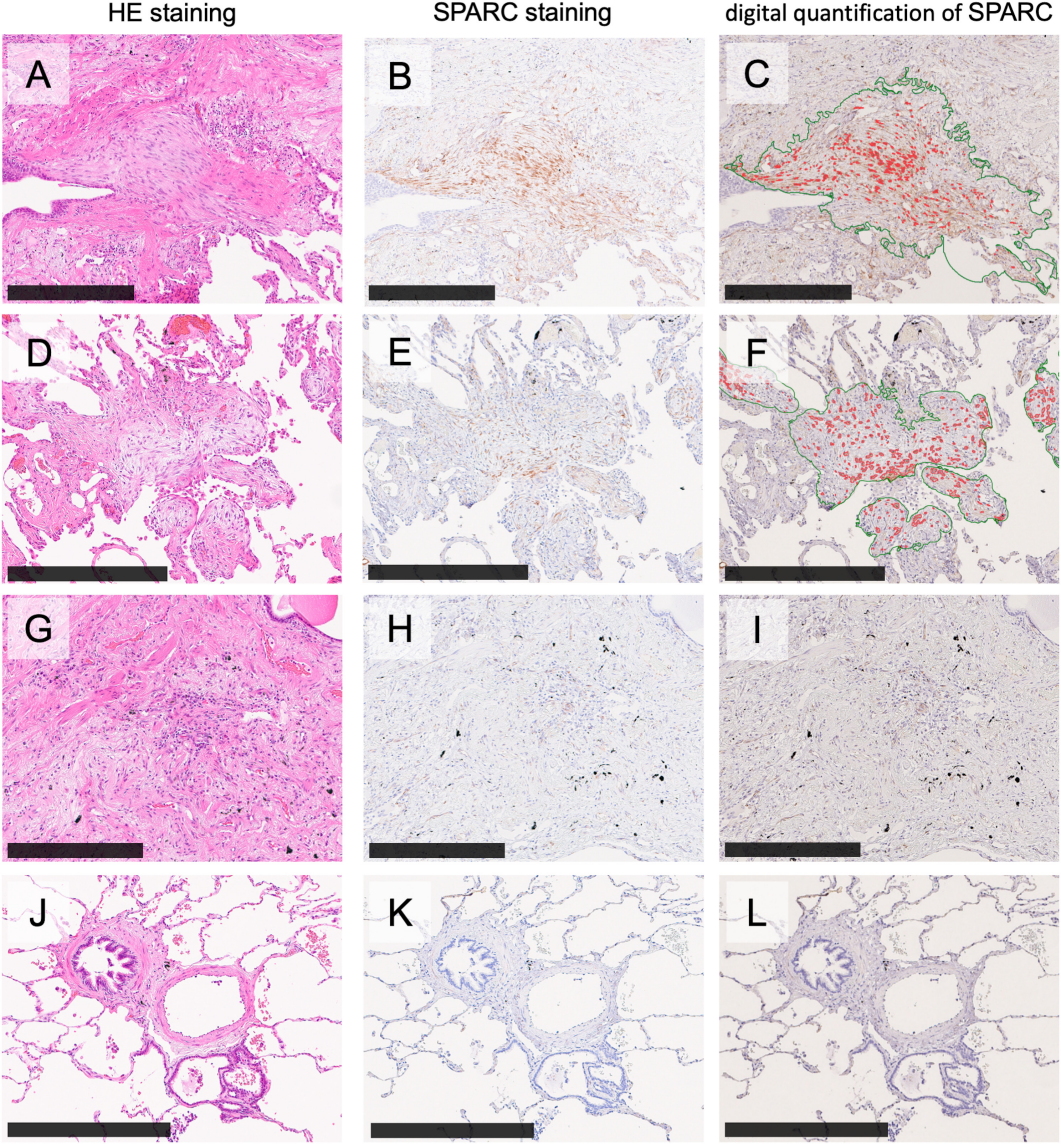
Immunohistochemical expression of SPARC in various pathological lesions and anatomical parts and digital quantification of its levels. Strong SPARC expression is observed in the cytoplasm of fibroblastic cells within (A–C) the fibroblastic focus and (D–F) destructive alveolar organization. However, SPARC expression is absent in (G–I) old scarring lesions and anatomically normal regions without pathological changes, such as (J–L) bronchioles and vessels. Immunohistochemical signals were automatically detected using a morphometric software (QuPath). The red overlay indicates SPARC‐positive cells, and the green overlay indicates SPARC‐positive areas. Panels show: (A, D, G, J) HE staining; (B, E, H, K) SPARC immunohistochemistry; (C, F, I, L) digital quantification for SPARC immunohistochemical signals. Scale bars, 400 μm.

To validate the clinical relevance of SPARC, we performed immunohistochemistry across various types of lesions. SPARC was expressed in fibroblastic foci (Figure 5A–C) and in destructive alveolar organization (Figure 5D–F). In contrast, SPARC was not expressed in tracheal old scarring lesions (Figure 5G–I) and vascular smooth muscles (Figure 5J–L).

### SPARC prognosis‐correlation analysis in uILDs

**Figure 6:**
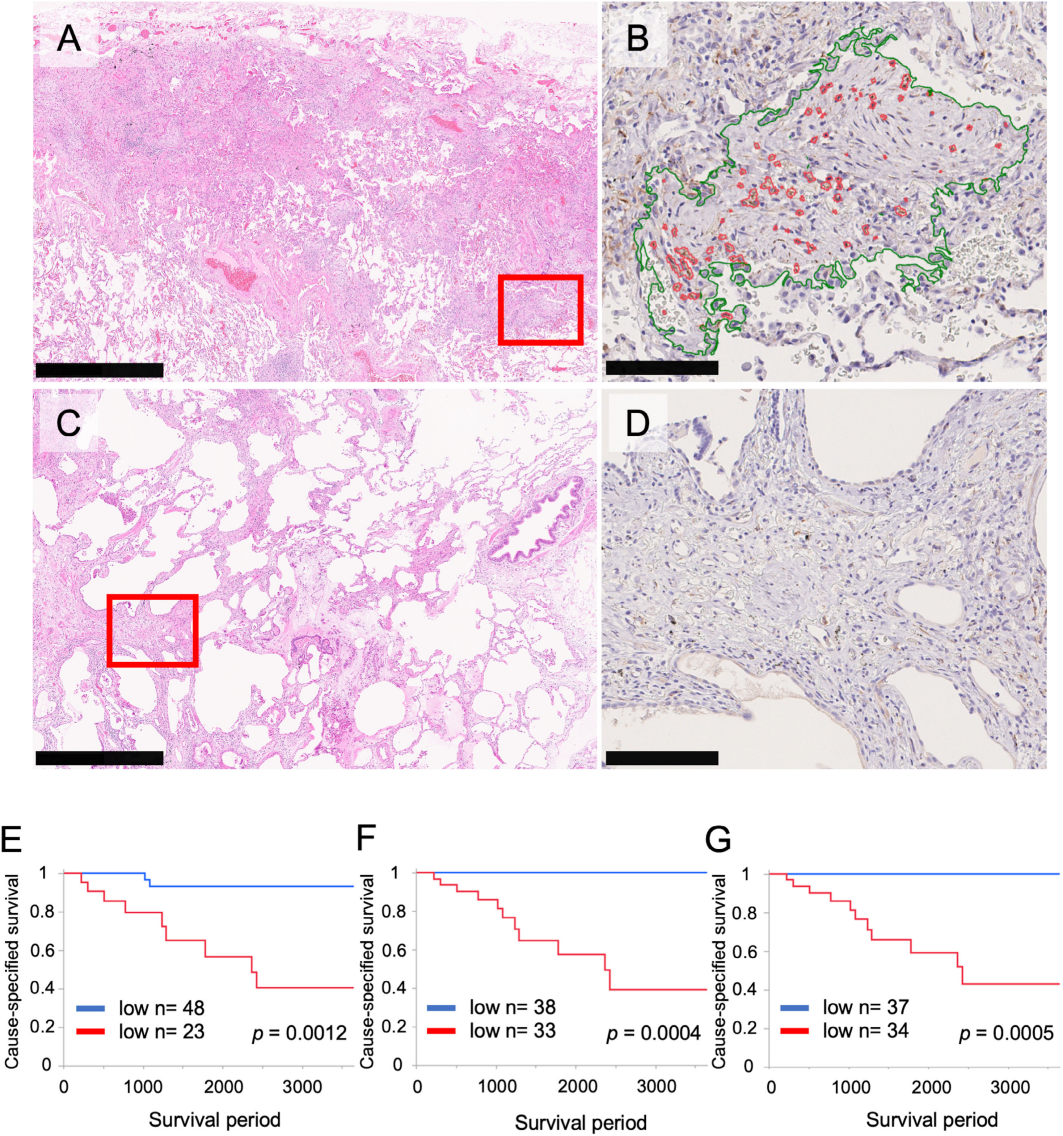
Immunohistochemical expression of SPARC in unclassifiable ILD (uILD). Histological appearance and SPARC immunohistochemistry in two representative uILD cases, which are (A, B) rich or (C, D) poor in young fibrotic lesions, are shown. Strong SPARC signals are seen in (A, B) fibroblastic cells in panels but not in (C, D) old scarring lesions/focal collapse fibrosis. Panels (A) and (C) are HE‐stained; panels (B) and D) show digital quantification of SPARC immunohistochemical signals. Scale bars, 1 mm (left), 100 μm (right). (E–G) Survival analyses correlating SPARC staining intensity with prognosis in unclassifiable ILD (uILD). SPARC rates from the upper and lower lobes, and the average of both, were calculated and cutoff values determined using receiver operating characteristic curves. Patients were divided into high and low SPARC expression groups based on these cutoffs for upper, lower, and combined lobes. Kaplan–Meier curves showing (A) upper, (B) lower, (C) average overall survival rates. The survival rate was significantly worse in the group in which SPARC showed high expression in any comparison. *p* values from the log‐rank test were as follows: panel (A), *p* = 0.0012; panel (B), *p* = 0.0004; panel (C), *p* = 0.0005. A *p* value <0.05 was considered statistically significant.

To further validate the prognostic potential of SPARC in ILDs, we extended our analysis to uILDs, a subgroup for which no established prognostic markers currently exist. SLB specimens from 71 patients with uILD underwent SPARC immunohistochemistry. Clinical characteristics of these patients are provided in the supplementary material, Table S1. The SPARC rate thresholds, determined using the maximum Youden index (see Materials and methods section), were 0.0134% for the upper lobe and 0.0092% for the lower and both lung lobes (supplementary material, Figure S6A–C). The corresponding area under the curve values were 0.790, 0.794, and 0.838, respectively, with high‐SPARC expression identified in 23, 33, and 34 cases. Histological examination showed that SPARC was markedly expressed in young fibrotic lesions, including in uILD cases (Figure 6A,B), whereas its expression was absent in old scarring lesions (Figure 6C,D).

Kaplan–Meier survival analysis demonstrated that patients with higher SPARC rates—whether in the upper, lower, or both lung lobes—had significantly worse prognoses (Figure 6E–G). Multivariate analysis adjusting for age, sex, and smoking history—potential confounding factors associated with prognosis—further confirmed that SPARC rate is an independent prognostic indicator in patients with uILD (supplementary material, Table S2).

## Discussion

The purpose of this study was to explore common and essential molecules that promote the fibrotic process, which can be a sign reflecting tissue injury and a marker for poor prognosis across a range of ILDs. Here, we focused on young fibrotic lesions, including fibroblastic foci and destructive alveolar organization, as both are characterized by fibroblastic aggregation and are widely recognized as indicators of disease progression and poor prognosis.

Our analysis identified a distinct SM2 gene module enriched in these early lesions—termed ‘young fibrotic lesions’—which are characterized by an interconnected network of hub genes including *COL1A1, COL1A2, COL3A1*, and *SPARC*. An integrative analysis using public scRNA‐seq data supported that the top hub genes of the SM2 module identified in the ST analysis may be involved in fibrosis. Furthermore, immunohistochemical validation confirmed that SPARC is upregulated in young fibrotic lesions and is strongly associated with poor prognosis in patients with uILD. These findings underscore the central role of SPARC and its related network in the fibrotic process, extending our understanding of fibrosis beyond traditional histological boundaries.

To explore common and essential molecules promoting fibrosis, understanding how these lesions are spatially organized within lung tissue is crucial. However, scRNA‐seq, which isolates cells for individual analysis, does not preserve the spatial dynamics [30], making it difficult to capture the structural relationships between fibrotic lesions and their microenvironment.

To address this, we initially focused on young fibrotic lesions, defined by fibroblastic foci and destructive alveolar organization, both of which are associated with poor prognosis and steroid nonresponsiveness [31]. Next, by applying hdWGCNA and the meta‐spots grouping technique within established ST platforms, we aimed to overcome the resolution limitations of traditional ST analysis [10]. This approach enhances the spatial resolution of transcriptomic data, allowing a more precise characterization of young fibrotic lesions. Our study provides new insights into how these markers are expressed within the tissue architecture, reinforcing their pathological and clinical significance.

Building on our findings, we focused on SPARC, a multifunctional regulator of fibrogenesis. SPARC interacts with collagen, facilitates procollagen processing, and plays a key role in ECM assembly [32]. It has also been implicated in TGF‐β‐induced fibroblast activation, apoptosis resistance, and paracrine signaling that disrupts alveolar epithelial barrier integrity and increases permeability [33, 34, 35]. Our single‐cell analysis further suggests that SPARC‐positive fibroblasts may closely interact with aberrant basaloid cells, which are thought to be involved in lung fibrogenesis [36], thereby supporting the established paracrine roles of SPARC (supplementary material, Figure S5B–D). These previous findings support our notion that SPARC can be one key molecule to form young fibrotic lesions at injured sites, which encouraged us to examine a large cohort for its potential prognostic value.

While recent studies have highlighted the need for biomarkers to predict disease progression across various ILD subtypes, particularly when conventional clinical parameters fall short [35], emerging evidence suggests that molecular signatures may be more reliable than clinical or radiological features in predicting outcomes for patients with progressive fibrosis [37].

Given the potential prognostic role of SPARC in fibrotic lung diseases, we sought to explore its clinical relevance in patients with uILD, a particularly challenging ILD subset. uILD is characterized by fibrotic forms with nonspecific clinical and radiological features and poor prognoses. Despite MDD, 10–20% of patients with ILD remain without a definitive diagnosis [38, 39, 40]. uILD remains a ‘black box’ owing to the lack of standardized diagnostic criteria [41], making its management particularly challenging. However, young fibrotic lesions have been suggested to be associated with prognosis even in patients with uILD [25].

Our large‐scale cohort study suggests a significant association between the proportion of SPARC‐stained fibroblasts and prognosis in patients with uILD, highlighting SPARC as a potential biomarker. While further research is needed to elucidate the underlying mechanisms, these findings suggest that targeting SPARC‐related pathways may offer a promising avenue for future therapeutic strategies [42].

Our study has several limitations. First, the limited number of patients and validated ROIs may affect the generalizability of our findings to larger populations. However, the cross‐disease validation process enhances the robustness of the results. Second, young fibrotic lesion‐specific gene expression patterns could be extracted using laser microdissection, as previously described [43]. The ST analysis profiling system aided in comparing distinct histological elements while preserving morphological context within a single tissue section, enabling comprehensive visualization, quantitative analysis, and enhanced intersample evaluation. Third, whether SPARC expression correlates with specific clinical phenotypes or strictly reflects treatment responsiveness in patients with uILD remains unclear. In addition, whether they can be fully distinguished in terms of disease progression remains unclear. Further clinically relevant forms of verification are needed. Fourth, SPARC is not a novel molecule; rather, it serves as a surrogate marker for fibroblasts, reflecting fibroblast density. Furthermore, distinguishing young fibrotic lesions from pure Masson bodies, which represent nondestructive alveolar organization, remains challenging from both pathological and biological perspectives. Indeed, SPARC expression has been reported in the Masson body, which is a sign of a favorable response to steroids [44]. However, by integrating transcriptomics and large‐cohort immunohistochemistry, our study achieved bridging genetic signatures with their pathological and clinical relevance, reaffirming the critical role of SPARC in patients with various ILDs. Finally, SPARC is a soluble protein and can be measured in serum, highlighting the need for further noninvasive and objective assessments of progressive fibrosis based on serum SPARC levels.

In conclusion, our study provided significant insights into common mechanisms of PF through unbiased multimodal transcriptome analysis. Moreover, we established SPARC as a key prognostic marker in patients with ILDs, highlighting its potential role in guiding future therapeutic strategies.

## Author contributions statement

KF, YN, KO and TO designed the study. TK, KF, DM and KO performed the experiments. TN and TK analyzed the data with the technical support of DM and SS. HK, TN, TB and TO, who collected the human clinical samples. TN and TK wrote the original draft. KF, DM, DO and KO provided helpful discussions. KF and KO reviewed and edited the article. KF, SS, SA, KO and AK supervised the study. TO acquired the research grants and funding.

## Supporting information


Supplementary materials and methods
**Figure S1**. Hematoxylin and eosin (HE) staining, Alcian blue‐periodic acid Schiff (Alb‐PAS) staining, and Elastica van Gieson (EVG) staining in young fibrotic lesions (YFLs)
**Figure S2**. Gene ontology (GO) enrichment analysis of genes in the SM2 module identified *via* hdWGCNA
**Figure S3**. Identification of SM2 module‐enriched cell populations using public single‐cell RNA sequencing data (GSE135893)
**Figure S4**. Trajectory‐based differential expression analysis of SM2 module‐enriched fibroblast populations using public scRNA‐seq data (GSE135893)
**Figure S5**. Cell–cell communication analysis of fibroblast subtypes using single‐cell RNA sequencing data
**Figure S6**. ROC curve based on the SPARC rate
**Table S1**. Clinical and pathological profiles of patients with uILD
**Table S2**. Multivariate analysis of correlations between prognosis and clinicopathological profiles

## Data Availability

Additional data supporting the conclusions of this study can be obtained from the corresponding author upon reasonable request. The raw sequencing data from this study were deposited in the NCBI GEO accession number: GSE264233 (https://www.ncbi.nlm.nih.gov/geo/query/acc.cgi?acc=GSE264233 [accessed on 04/June/2025]). The data are currently available through reviewer token access and will be made publicly accessible prior to the proof stage. All code is available at https://github.com/mosatsu/spatial-and-scRNA-seq-analysis-for-patients-with-pulmonary-fibrosis.
