# Supplementary material for: Spatial transcriptomics identifies SPARC as a prognostic marker in interstitial lung diseases
Source: J Pathol. 2025 Jul 28;267(1):79–91. doi: 10.1002/path.6451 (PMC12337812; doi:10.1002/path.6451)
Supplement: Supplementary file 1 — Supplementary materials and methods Figure S1. Hematoxylin and eosin (HE) staining, Alcian blue‐periodic acid Schiff (Alb‐PAS) staining, and Elastica van Gieson (EVG) staining in young fibrotic lesions (YFLs) Figure S2. Gene ontology (GO) enrichment analysis of genes in the SM2 module identified via hdWGCNA Figure S3. Identification of SM2 module‐enriched cell populations using public single‐cell RNA sequencing data (GSE135893) Figure S4. Trajectory‐based differential expression analysis of SM2 module‐enriched fibroblast populations using public scRNA‐seq data (GSE135893) Figure S5. Cell–cell communication analysis of fibroblast subtypes using single‐cell RNA sequencing data Figure S6. ROC curve based on the SPARC rate Table S1. Clinical and pathological profiles of patients with uILD Table S2. Multivariate analysis of correlations between prognosis and clinicopathological profiles [file PATH-267-79-s001.docx]

**Spatial transcriptomics identifies SPARC as a prognostic marker in interstitial lung diseases**

T Niitsu, T Kataoka, K Fukushima *et al.* *J Pathol* <https://doi.org/10.1002/path.6451>

**Supplementary materials and methods**

**Supplementary Figures S1****–S6**

**Supplementary Tables S1, S2**

Reference numbers refer to the main text list.

**Supplementary materials and methods**

**GO enrichment analysis for the extracted module from spatial transcriptomics data**

To investigate functional enrichment of gene expression modules extracted from spatial high-dimensional weighted gene co-expression network analysis (hdWGCNA), we employed the EnrichR package, a tool that facilitates gene set enrichment analysis across multiple databases. We selected two Gene Ontology (GO) databases—GO_Biological_Process_2021 and GO_Molecular_Function_2021 [https://maayanlab.cloud/Enrichr/#libraries; last accessed 15/February/2025]—to assess the functional relevance of genes identified in our analysis. Enrichment analysis was performed using the RunEnrichr function in hdWGCNA, with a maximum of 100 genes per module considered for testing. To visualize the enriched GO terms, bar plots were created using EnrichrBarPlot, displaying the top 10 enriched terms for each module.

**Single-cell RNA sequencing analysis and extracted module enrichment scoring**

The publicly available scRNA-seq data from patients with different forms of pulmonary fibrosis (PF) were analyzed, including idiopathic pulmonary fibrosis (IPF) (*n* = 12), chronic hypersensitivity pneumonitis (cHP) (*n* = 3), nonspecific interstitial pneumonia (NSIP) (*n* = 2), unclassifiable interstitial lung disease (uILD) (*n* = 1), and nonfibrotic controls (*n* = 10) (GSE135893; <https://www.ncbi.nlm.nih.gov/geo/query/acc.cgi?acc=GSE135893>) [26]. The data were pre-processed using the R Seurat package to identify 30 principal components and define cell clusters at a resolution of 1. Uniform Manifold Approximation and Projection (UMAP) algorithm [45] was applied to project high-dimensional data onto a two-dimensional space for visualization, revealing distinct Seurat-defined clusters. Subsequently, re-clustering was performed on the extracted module enrichment Cluster  to identify 20 principal components and define cell clusters at a resolution of 0.5.

The SM2 module, consisting of the top 20 hub genes (*SPARC*, *COL1A1*, *MMP2*, *THBS2*, *CERCAM*, *FBN1*, *TNC*, *POSTN*, *LUM*, *FKBP10*, *CTHRC1*, *COL3A1*, *COL1A2*, *COL6A3*, *COL6A2*, *COL5A1*, *COL16A1*, *COL5A2*, *DCN*, and *COL6A1*), was used to calculate enrichment scores using <AddModuleScore()> from the R Seurat package and <enrichIt()> from the R UCell package. UMAPs were generated using <featurePlot()> to visualize SM2 enrichment across cell types. Trajectory-based differential expression analysis was performed to assess SM2 module genes across pseudotime, using <AddModuleScore()> from the R Seurat package and <enrichIt()> from the R UCell package. The UMAP results were visualized using <featurePlot()>.

**Trajectory-based differential expression analysis for single-cell sequencing data**

As described above, the original and publicly available (GSE135893; https://www.ncbi.nlm.nih.gov/geo/query/acc.cgi?acc=GSE135893) scRNA-seq data were used, and reclustering was performed. The R Slingshot package was used to infer developmental trajectories and pseudotime. Enrichment scores were calculated for each Cluster using the featureplot and AddModulesScore functions from the Seurat package. Cluster 1 was defined as the starting point for the trajectory analysis.

To identify the differentially expressed genes along the trajectories [28], we used the fitGAM function of tradeSeq with 3–20 evaluated knots. A total of 20 knots was found to strike the best balance between model flexibility and overfitting risk. Random seeds were used to ensure reproducibility across the analyses.

We used the startVsEndTest function to discover the progenitor marker genes and the associationTest function to test whether the average gene expression substantially changed along pseudotime. The patternTest function assessed whether the smoothed gene expression remained consistent along pseudotime across multiple lineages, serving as an indicator of whether there are differences in gene expression patterns between lineages. The top 10 retrieved genes from the patternTest are listed in supplementary material, Figure S4C. The diffEndTest function was used to identify differentiated cell-type markers among lineage comparisons (supplementary material, Figure S4D).

**Cell-cell communication analysis for single-cell sequencing data**

To construct the cell-cell communication network from SM2 module-enriched cell types in the fibrotic niche, we utilized the CellChat [29] package to predict ligand-receptor interactions between different cell populations using GSE135893 scRNA-seq data. Cell-cell communication networks were visualized using <netVisual_circle()>, generating two plots; one plot depicting the top 10 cell types based on the number of interactions and another based on interaction strength. To focus on biologically relevant interactions, enriched signaling pathways were identified using <extractEnrichedLR()>. Their contributions to receptor-expressing cell communication were further quantified using <netAnalysis_contribution()>.

Chord diagrams were generated using <netVisual_aggregate()> to illustrate the signaling pathways contributing to intercellular communication. Additionally, the most significant ligand-receptor pairs within the most enriched pathway were identified to determine those with the strongest contributions to intercellular communication. These pairs were visualized as chord diagrams generated with <netVisual_chord_gene()>.


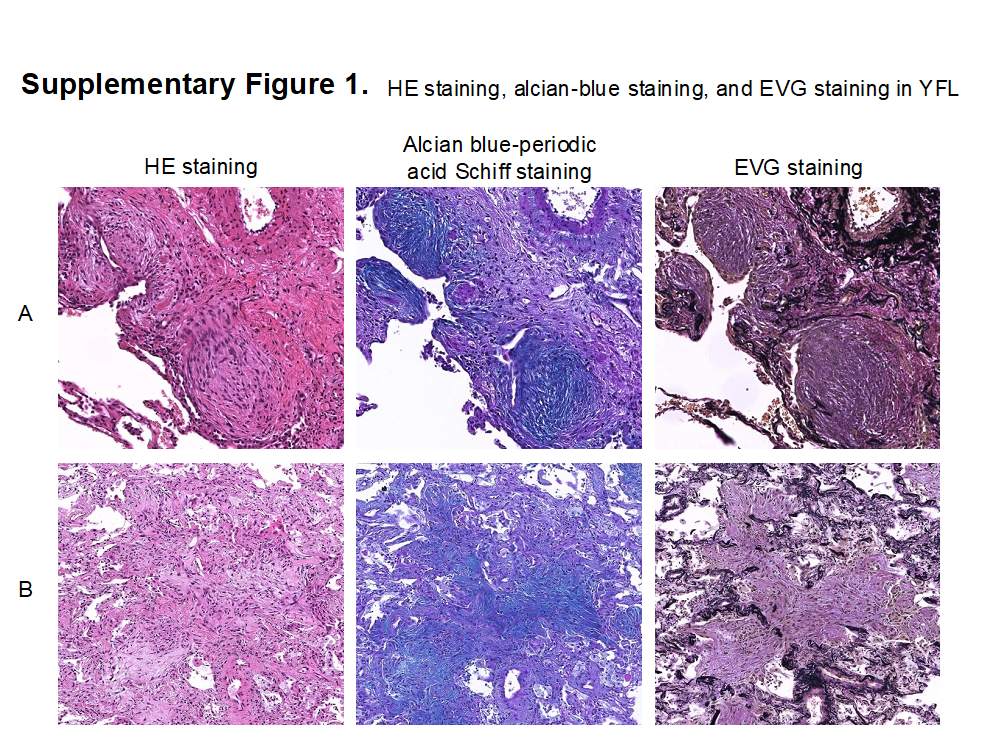


**Figure S1. Hematoxylin and eosin (HE) staining, Alcian blue-periodic acid Schiff (AB-PAS) staining, and Elastica van Gieson (EVG) staining in young fibrotic lesions. (YFLs)**. HE staining, AB-PAS staining, and EVG staining in (A) fibroblastic foci (FF) and (B) destructive alveolar organization. Fibroblastic cells in YFLs were stained light blue and were well visualized by AB-PAS staining. Scale bars, 300 μm.


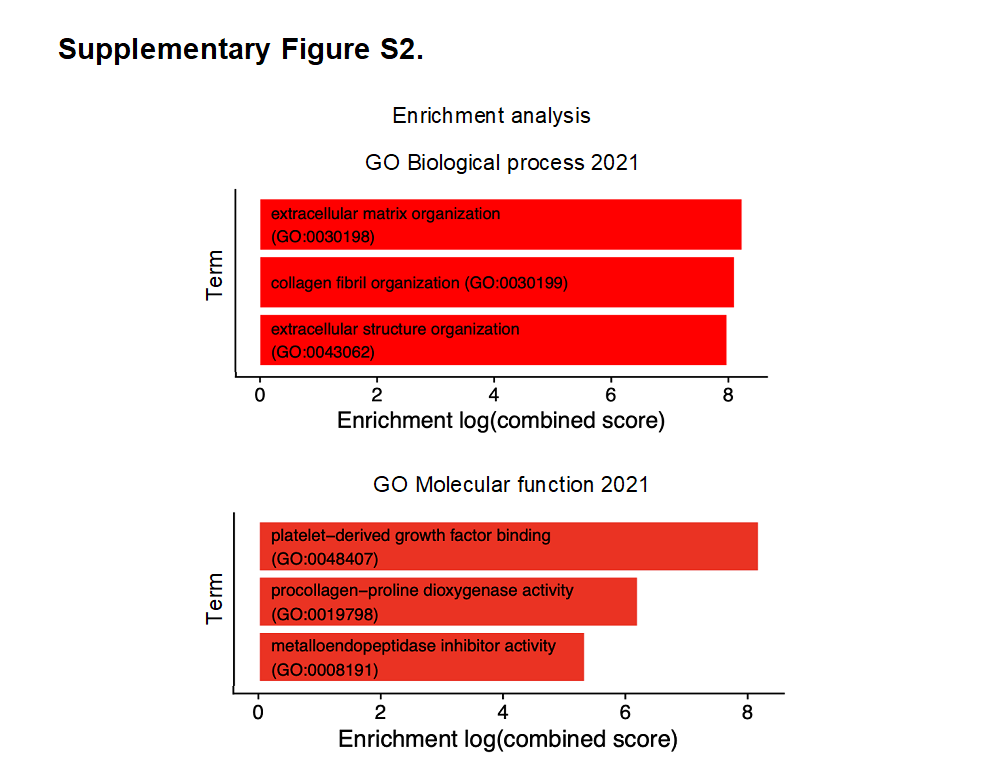


**Figure S2. Gene ontology (GO) enrichment analysis of genes in the SM2 module identified using hdWGCNA.** The top enriched GO terms are shown for two categories: (A) GO Biological Process 2021 and (B) GO Molecular Function 2021. Each bar represents a specific biological process, with the length of the bar corresponding to the enrichment score or statistical significance of the process. The analysis was performed using the RunEnrichr function implemented in the R packages hdWGCNA and enrichR.


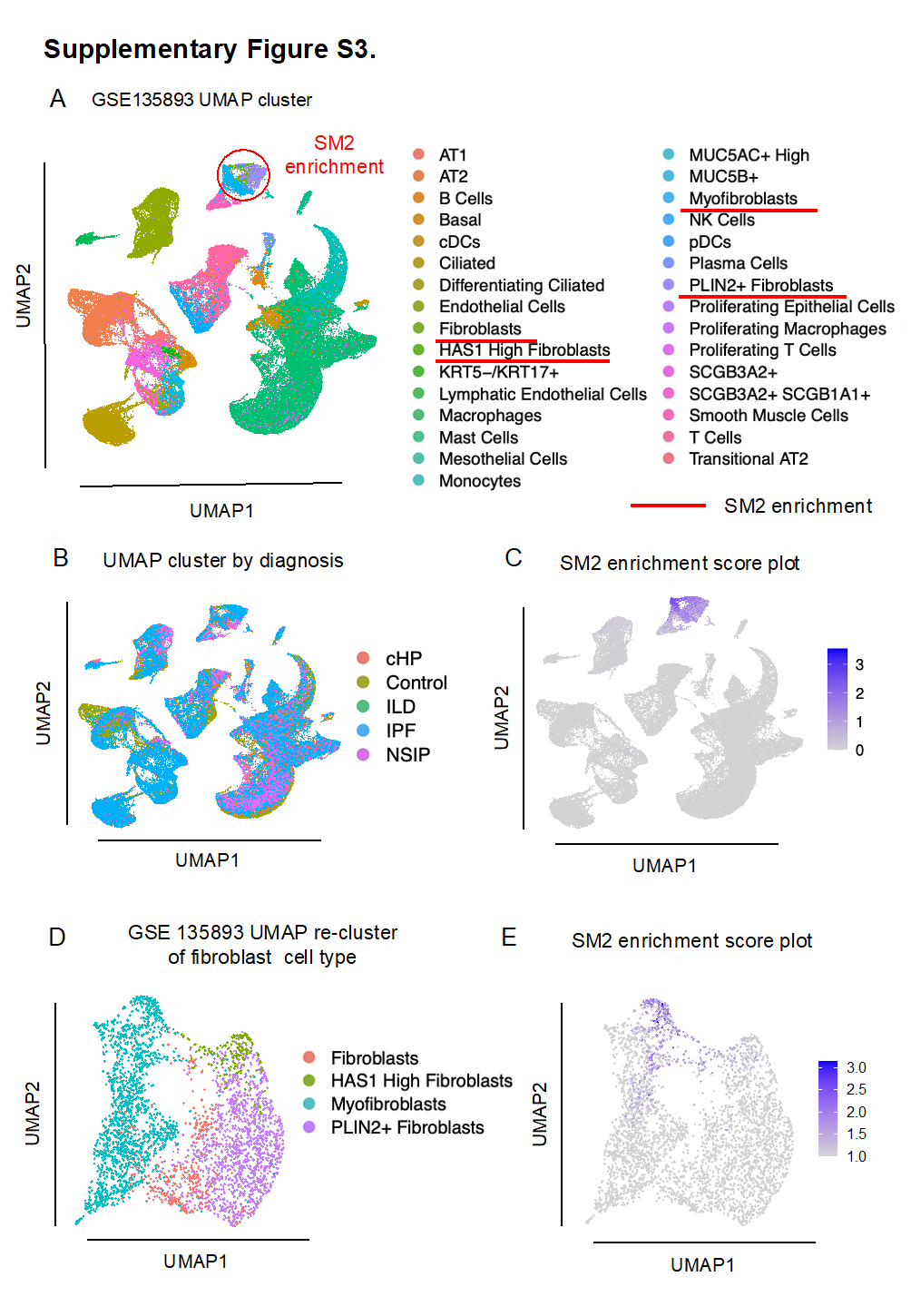


**Figure S3. Identification of SM2 module-enriched cell populations using public single-cell RNA sequencing data (GSE135893)**. (A) UMAP visualization of single-cell transcriptomes (GSE135893), annotated by cell type. (B) UMAP colored by diagnosis, including chronic hypersensitivity pneumonitis, interstitial lung disease, idiopathic pulmonary fibrosis, nonspecific interstitial pneumonia, and control. (C) Feature plot showing the enrichment score of the SM2 module across all cell types. Enrichment is localized to distinct fibroblast populations. (D) UMAP re-clustering of fibroblast populations, further subclassified into Fibroblasts, HAS1 High Fibroblasts, Myofibroblasts, and PLIN2+ Fibroblasts. (E) SM2 module enrichment score plot showed strongest enrichment in HAS1 High Fibroblasts and Myofibroblasts.


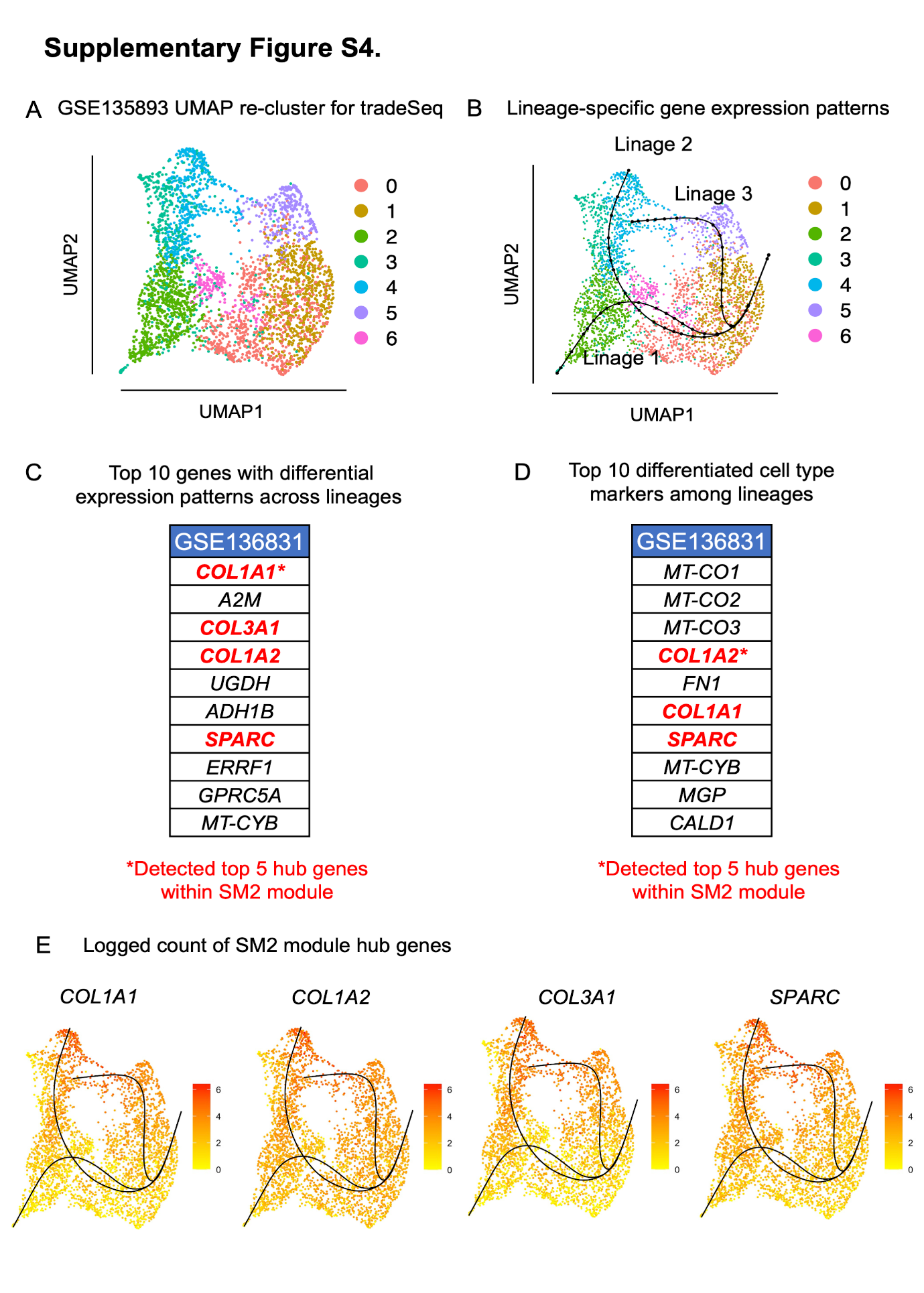


**Figure S4. Trajectory-based differential expression analysis of SM2 module-enriched fibroblast populations using public scRNA-seq data (GSE135893). (**A) UMAP re-clustering of fibroblast populations used for pseudotime trajectory inference by Slingshot. Cluster 1 was defined as the starting point. The re-clustering was performed with dimensionality set to 20 and resolution set to 0.5 using Seurat. (B) Lineage-specific gene expression trajectories inferred from the pseudotime analysis. Three distinct lineages were identified: Lineage 1: from PLIN2+ Fibroblasts to Myofibroblasts enriched in CTHRC1—a SM2 module-enriched trajectory; Lineage 2: from PLIN2+ Fibroblasts to HAS1⁺ High Fibroblasts—also enriched for SM2 module genes; Lineage 3: from PLIN2+ Fibroblasts to Myofibroblasts enriched in quiescence-associated genes such as INMT and NPNT. (C) Top 10 genes showing differential expression patterns across the three lineages. SM2 hub genes (*COL1A1*, *COL3A1*, *COL1A2*, *SPARC*) are highlighted in red. (D) Top 10 cell type marker genes that distinguish the three lineages. SM2 hub genes also appear among these markers. (E) Expression feature plots showing log-normalized expression of SM2 module hub genes (*COL1A1*, *COL1A2*, *COL3A1*, *SPARC*) across the fibroblast lineages.


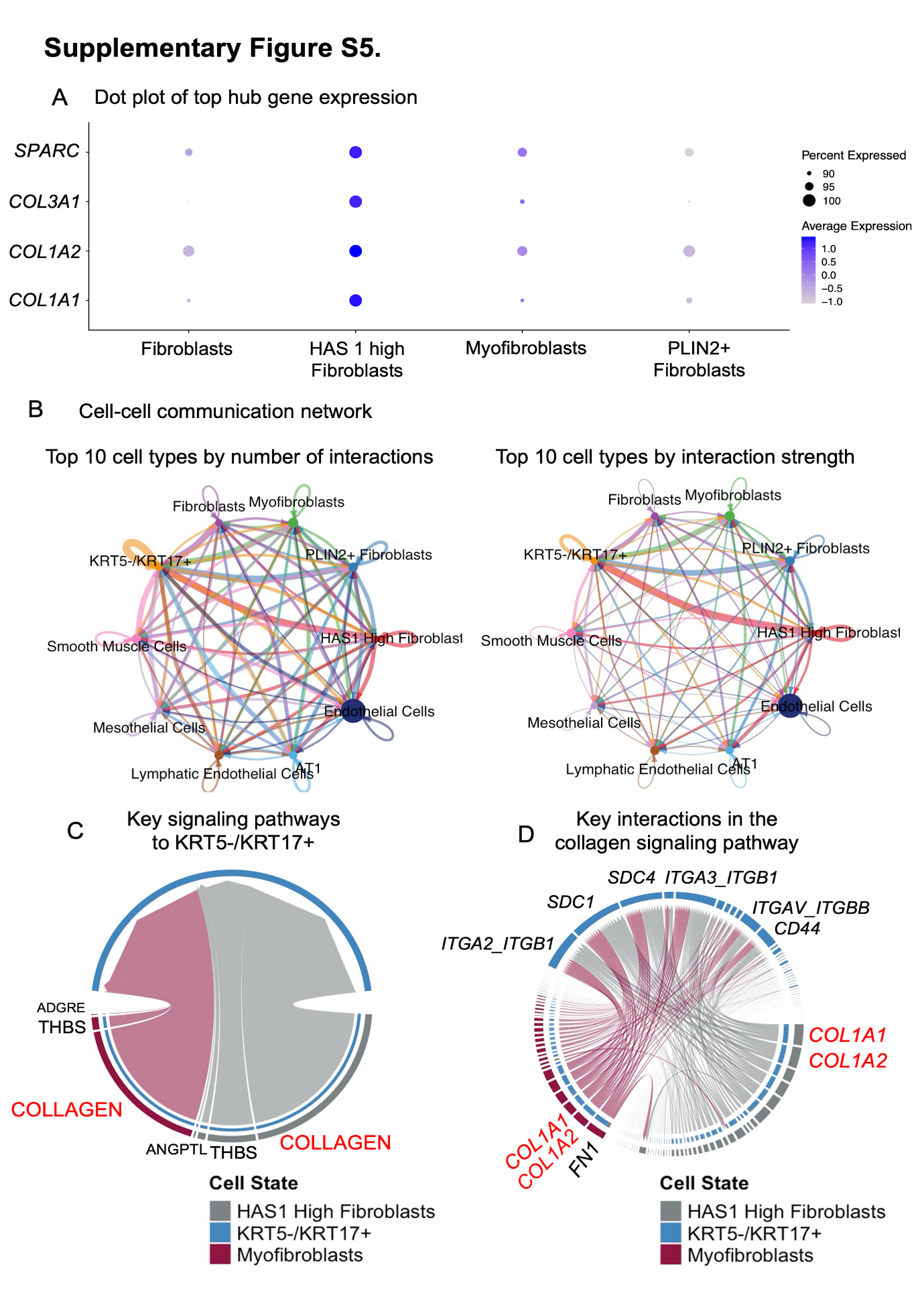


**Figure S5. Cell-cell communication analysis of fibroblast subtypes using single-cell RNA sequencing data. (**A) Dot plot showing expression of SM2 module top hub genes (*COL1A1*, *COL1A2*, *COL3A1*, *SPARC*) across fibroblast subtypes: Fibroblasts, HAS1 High Fibroblasts, Myofibroblasts, and PLIN2+ Fibroblasts. (B) Cell-cell communication network analysis showing the top 10 cell types ranked by (left) number of inferred interactions and (right) overall interaction strength. Fibroblast subtypes, particularly HAS1 High Fibroblasts and Myofibroblasts, exhibit high levels of intercellular communication. (C) Key signaling pathways contributing to communication with KRT5⁻/KRT17⁺ epithelial cells. The collagen signaling pathway is the most enriched among interactions originating from both HAS1 High Fibroblasts and Myofibroblasts. (D) Visualization of major ligand-receptor pairs within the collagen signaling pathway. Interactions involving COL1A1 and COL1A2 are highly prominent in both HAS1 High Fibroblasts and Myofibroblasts.


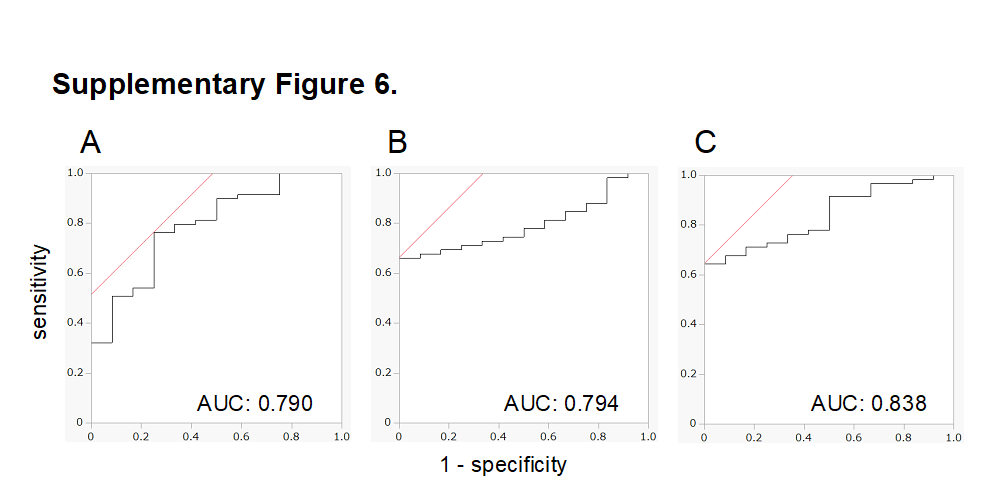


**Figure S6. ROC curve based on the SPARC rate.** ROC curve based on the SPARC rate of (A) the upper lobe, (B) the lower lobe, and (C) both lung lobes. Sensitivity is presented on the vertical axis, and 1-Specificity is presented on the horizontal axis.

**Table S1.** Clinical and pathological profiles of patients with uILD.

|  | ***n*=71 patients with uILD** |
| --- | --- |
| **Sex** |  |
| Female | 27 |
| Male | 44 |
| **Age (yrs)** |  |
| >65 | 39 |
| ≦65 | 32 |
| Mean (SD) | 64.8 (6.8) |
| **Smoking** |  |
| PYI >20 | 34 |
| PYI ≦20 | 37 |

PYI, package-year index; SD, standard deviation; uILD, unclassifiable interstitial lung disease.

**Table S2.** Multivariate analysis of correlations between prognosis and clinicopathological profiles.

|  | ***p*-value** |
| --- | --- |
| **Upper lobe** |  |
| Sex | 0.5317 |
| Age | 0.9844 |
| Smoking | 0.8789 |
| SPARC rate | 0.0012 |
| **Lower lobe** |  |
| Sex | 0.9888 |
| Age | 0.5845 |
| Smoking | 0.952 |
| SPARC rate | <0.0001 |
| **Both lobes** |  |
| Sex | 0.8056 |
| Age | 0.7293 |
| Smoking | 0.7882 |
| SPARC rate | <0.0001 |

SPARC rate, the total SPARC-positive area among young fibrotic lesions divided by the sample area for each case.
